# Diagnostic Complexity in Systemic Inflammation: Adult-Onset Still’s Disease

**DOI:** 10.7759/cureus.101864

**Published:** 2026-01-19

**Authors:** Kevin Rivera, Caitlin Kesari

**Affiliations:** 1 Internal Medicine, Mount Carmel Health System, Columbus, USA; 2 Rheumatology, Mount Carmel Health System, Columbus, USA

**Keywords:** adult-onset still’s disease, autoinflammatory disease, hyperferritinemia, il-1 inhibitor therapy, yamaguchi criteria

## Abstract

Adult-onset Still’s disease (AOSD) is a rare systemic autoinflammatory disorder classically associated with fever, rash, arthritis, and marked inflammatory laboratory abnormalities. Diagnosis is clinical and typically requires exclusion of infection, malignancy, and other rheumatologic diseases. We describe a 33-year-old previously healthy Nigerian male with several weeks of daily fevers by history, diffuse polyarthralgia with synovitis, sore throat, and a transient non-pruritic rash initially treated as an allergic reaction. His symptoms progressed despite antibiotics. Outpatient testing showed ferritin >20,000 ng/mL and CRP 328 mg/L. During admission, he had persistent leukocytosis (29-30×10⁹/L) and hyperferritinemia (5,770-14,000 ng/mL). Knee aspiration revealed calcium pyrophosphate crystals consistent with calcium pyrophosphate dihydrate (CPPD), which added diagnostic uncertainty regarding whether a crystal arthropathy was driving his syndrome or represented a coincident finding. He received empiric broad-spectrum antimicrobials early in the hospitalization for possible severe infection, but clinical improvement occurred only after high-dose corticosteroids. Given profound hyperferritinemia, hemophagocytic lymphohistiocytosis and macrophage activation syndrome were considered, although cytopenias were absent and bone marrow biopsy showed no hemophagocytosis. Rheumatology diagnosed AOSD using classification criteria applied in a clinical context after exclusion of alternative etiologies. He improved with corticosteroids and later transitioned from anakinra to canakinumab with methotrexate due to suboptimal response and pruritus, with marked clinical improvement. This case highlights how CPPD crystal detection can complicate the interpretation of inflammatory arthritis in a patient whose overall presentation suggests systemic autoimmune inflammation and the importance of reassessing the unifying diagnosis when clinical features do not align.

## Introduction

Adult-onset Still’s disease (AOSD) is a rare systemic inflammatory disorder characterized by quotidian fevers, inflammatory arthritis, evanescent rash, and marked elevation of inflammatory markers [1]. It is increasingly recognized as part of a broader Still’s disease spectrum that includes systemic juvenile idiopathic arthritis and elderly-onset Still’s disease, with shared clinical and immunologic features across age groups [2]. Despite improved understanding of its immunopathology, AOSD remains a diagnostic challenge because its presenting features overlap with infection, malignancy, and other inflammatory rheumatologic conditions [3].

The incidence of AOSD is estimated at approximately 0.16 to 0.4 cases per 100,000 persons annually, with equal sex distribution and a bimodal age pattern affecting young and middle-aged adults [4]. The pathogenesis is thought to involve dysregulated innate immune activation with excessive production of pro-inflammatory cytokines, particularly interleukin (IL)-1, IL-6, IL-18, tumor necrosis factor alpha, and interferon gamma [1,5]. This cytokine-driven inflammation underlies both the systemic manifestations and laboratory abnormalities characteristic of the disease, including leukocytosis and hyperferritinemia [6].

Because no single diagnostic test exists, AOSD is a diagnosis of exclusion. Classification criteria, most commonly the Yamaguchi and Fautrel criteria, are frequently used to support diagnosis in clinical practice [7,8]. However, both sets of criteria rely on clinical features that may be incompletely documented or altered by prior treatment, particularly antipyretics and corticosteroids. As a result, real-world diagnosis often requires integrating clinical pattern recognition, excluding alternative etiologies, and longitudinal reassessment rather than rigidly applying criteria alone [3,9]. Hyperferritinemia is a prominent laboratory feature of AOSD and may be extreme, sometimes exceeding 10,000 ng/mL [10]. While elevated ferritin supports the diagnosis, it is not specific. It can prompt concern for macrophage activation syndrome (MAS) or hemophagocytic lymphohistiocytosis (HLH), both of which carry significant morbidity and mortality if unrecognized [11]. Distinguishing severe systemic inflammation from true MAS remains a frequent clinical dilemma.

Management of AOSD is guided by disease severity and phenotype. Corticosteroids remain first-line therapy for systemic inflammation, but many patients require escalation to disease-modifying therapy due to steroid dependence or refractory disease [5,12]. Biologic agents targeting IL-1 and IL-6 have substantially improved outcomes and are now central to treatment algorithms, particularly for systemic disease [6,13]. Despite these advances, diagnostic delays and misattribution of symptoms remain common, especially when competing diagnoses partially explain aspects of the presentation [14].

We present a case of AOSD in a young adult whose diagnostic course was complicated by the detection of calcium pyrophosphate dihydrate (CPPD) crystals on joint aspiration. AOSD classically presents with quotidian fevers, inflammatory arthritis, and a transient salmon-colored rash. It may be complicated by MAS or HLH, which represent life-threatening hyperinflammatory states. This case highlights how crystal-proven CPPD, although objectively confirmed, can coexist with and distract from recognition of an underlying systemic autoinflammatory disease, illustrating the need to interpret localized findings within the context of a broader inflammatory syndrome.

## Case presentation

A 33-year-old previously healthy Nigerian male presented with several weeks of daily fevers by history, diffuse polyarthralgia, and a transient non-pruritic rash. Three weeks prior to admission, he was evaluated at an emergency department for rash and malaise and was treated with corticosteroids, famotidine, and diphenhydramine for a presumed allergic reaction. One week later, he presented to a second emergency department with worsening arthralgias, myalgias, sore throat, and fever (101.1°F). Laboratory evaluation at that time demonstrated leukocytosis, mildly elevated lactate, and mild acute kidney injury, with urinalysis notable for trace hematuria. COVID-19 and streptococcal testing were negative, and the chest radiograph was unremarkable. He was discharged on amoxicillin for a presumed infection. Blood cultures were negative, and the urine culture grew *Enterococcus faecalis*, which was sensitive to amoxicillin.

Despite completing antibiotics, his joint symptoms progressed. He followed up with his primary care physician five days prior to admission, reporting constant and worsening polyarthralgia with joint swelling and impaired mobility. Laboratory testing demonstrated leukocytosis, anemia, and markedly elevated inflammatory markers. Autoimmune evaluation was unrevealing, including negative antinuclear antibody, rheumatoid factor, and HLA-B27 testing. He was referred to rheumatology and seen two days later. He appeared ill and required a wheelchair due to significant polyarthritis. Clinical examination documented synovitis involving the bilateral wrists, fingers, knees, and ankles, with reduced range of motion and impaired grip strength. Prednisone 60 mg daily was initiated empirically. Fevers were reported as daily, predominantly evening spikes associated with malaise, though high-grade temperatures were inconsistently documented during hospitalization. The rash, described earlier in the course, was transient, non-pruritic, and self-resolving, without clear temporal correlation to fever during inpatient observation. Outpatient testing revealed markedly elevated ferritin and inflammatory markers with mild transaminitis, prompting direct admission for further evaluation of a fever of unknown origin and systemic inflammation. Laboratory findings are summarized in Table 1.

**Table 1:** Summary of laboratory findings AST: aspartate aminotransferase, ALT: alanine aminotransferase, HIV: human immunodeficiency virus, COVID-19: coronavirus disease, CO₂: bicarbonate

Laboratory parameter	Reference range	Admission
White blood cell count	4.0-11.0 ×10⁹/L	29 ×10⁹/L
Hemoglobin	13.5-17.5 g/dL	10.9 g/dL
Platelet count	150-400 ×10⁹/L	440 ×10⁹/L
Erythrocyte sedimentation rate	0-15 mm/hr	96 mm/hr
C-reactive protein	<10 mg/L	328 mg/L
Ferritin	30-400 ng/mL	>20,000 ng/mL
Creatinine	0.7-1.3 mg/dL	1.38 mg/dL
Sodium	135-145 mmol/L	136 mmol/L
Potassium	3.5-5.0 mmol/L	4.5 mmol/L
Chloride	98-107 mmol/L	96 mmol/L
CO₂	22-29 mmol/L	30.0 mmol/L
Blood urea nitrogen	7-20 mg/dL	15 mg/dL
Glucose	70-99 mg/dL	78 mg/dL
Calcium	8.6-10.2 mg/dL	9.4 mg/dL
Total protein	6.4-8.3 g/dL	8.2 g/dL
AST	10-40 U/L	47 U/L
ALT	7-56 U/L	24 U/L
Alkaline phosphatase	44-147 U/L	84 U/L
Total bilirubin	0.2-1.2 mg/dL	0.5 mg/dL
Aldolase	≤8.1 U/L	5.8 U/L
Complement C3	82-185 mg/dL	138 mg/dL
Complement C4	15-33 mg/dL	35 mg/dL
Uric acid	3.4-7.0 mg/dL	Normal
Antinuclear antibody	Negative	Negative
Rheumatoid factor	Negative	Negative
HLA-B27	Negative	Negative
HIV	Negative	Negative
COVID-19	Negative	Negative
Blood cultures	No growth	No growth

**Figure 1:**
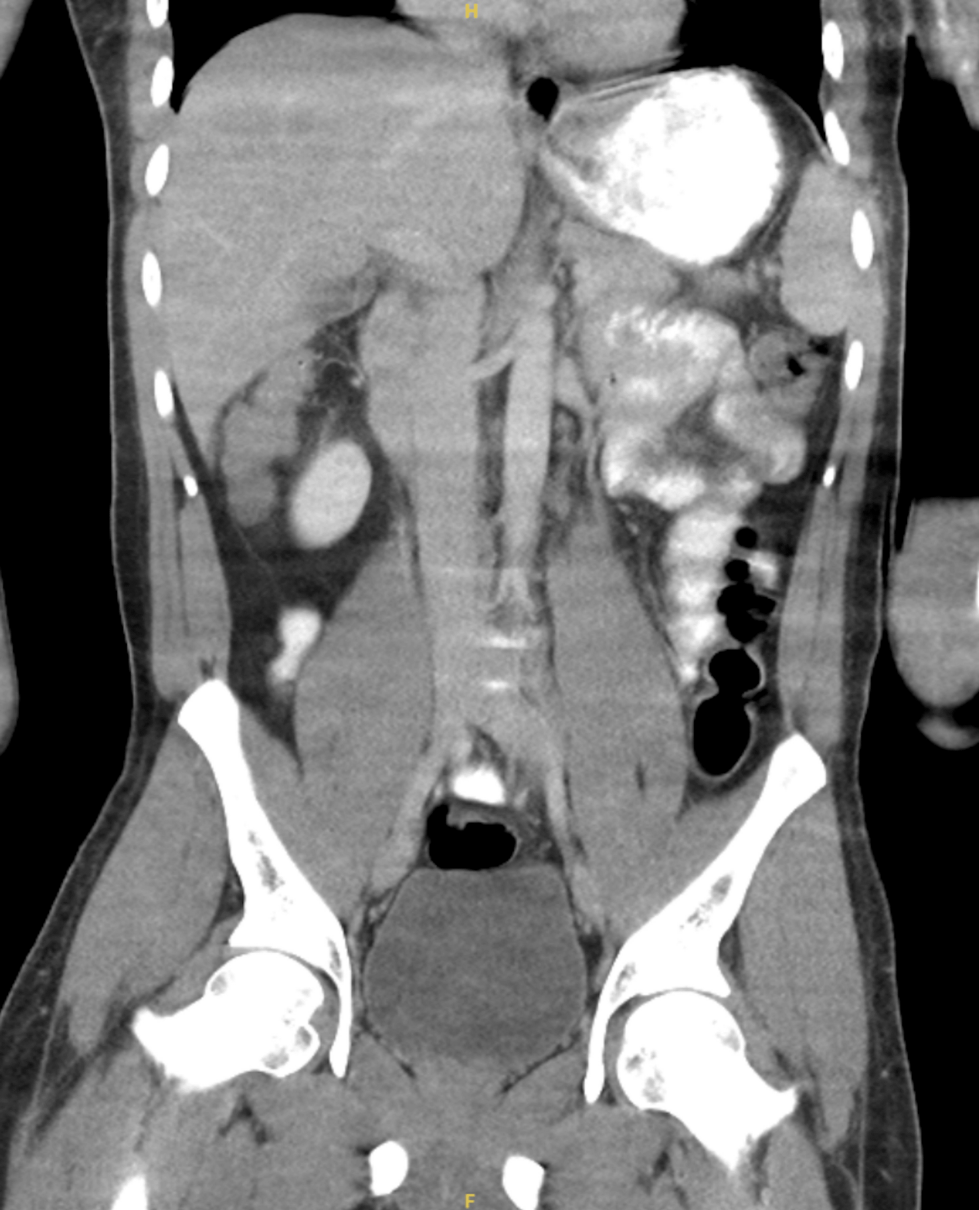
Coronal CT of the abdomen and pelvis demonstrating no acute intra-abdominal pathology, lymphadenopathy, splenomegaly, or mass lesion CT: computed tomography

On admission, he was febrile (Tmax 101.1°F), tachycardic (101 bpm), and ill-appearing with continued joint swelling and restricted mobility. Examination again documented synovitis in the wrists, fingers, knees, and ankles. No rash was present at the time of admission. Laboratory testing continued to show marked leukocytosis and hyperferritinemia, with normal uric acid. COVID-19 and HIV testing were negative, and blood cultures remained negative. Imaging, including computed tomography of the chest, abdomen, and pelvis, as well as magnetic resonance imaging of the spine, showed no acute pathology; notably, abdominal and pelvic imaging demonstrated no evidence of malignancy, lymphadenopathy, splenomegaly, or other alternative inflammatory drivers (Figure 1). The electrocardiogram and transthoracic echocardiogram were unremarkable. Knee joint aspiration performed by orthopedic surgery revealed calcium pyrophosphate crystals consistent with CPPD. CPPD is uncommon in younger adults and, in this context, was interpreted as a secondary finding rather than the primary driver of the patient’s systemic inflammatory syndrome.

Early in the hospitalization, he was empirically treated with broad-spectrum antimicrobials (daptomycin, meropenem, and doxycycline) for possible severe infection, given ongoing fever and systemic inflammation. He remained febrile until initiation of intravenous methylprednisolone 1 g daily for three days, followed by oral prednisone 60 mg daily. Fever and joint symptoms subsequently improved. Due to marked hyperferritinemia, HLH and MAS were considered. Bone marrow biopsy showed no hemophagocytosis or marrow suppression, and cytopenias were absent throughout admission.

He was discharged after six days. At outpatient follow-up, he reported improved mobility but persistent bilateral shoulder pain, managed with intra-articular glucocorticoid injections. He was initially started on daily anakinra but developed intermittent fevers and pruritus, prompting a transition to subcutaneous canakinumab and methotrexate. He reported marked improvement in systemic symptoms and joint function on this regimen. At outpatient follow-up after resolution of the acute flare, ferritin had normalized to 234 ng/mL. Rheumatology diagnosed AOSD based on fulfillment of classification criteria applied in a clinical context after exclusion of alternative etiologies.

## Discussion

This case illustrates the diagnostic complexity of AOSD when overlapping or partially explanatory findings complicate the interpretation of a systemic inflammatory presentation. The patient demonstrated a constellation of features classically associated with AOSD, including inflammatory polyarthritis with documented synovitis, daily fevers by history, sore throat, a transient rash earlier in the course, leukocytosis with neutrophilic predominance, transaminitis, and marked hyperferritinemia. Together, these findings reflect the cytokine-driven systemic inflammation characteristic of AOSD and align with established clinical descriptions of the disease [1,3,5].

Diagnostic criteria and clinical context

AOSD is a clinical diagnosis supported by characteristic features and the exclusion of alternative etiologies. Widely used classification frameworks emphasize the presence of quotidian fever, inflammatory arthritis, a transient salmon-colored rash, leukocytosis with neutrophilic predominance, and the absence of infectious, malignant, or other autoimmune causes [11]. In practice, applying these criteria often requires clinical judgment, particularly when features are incompletely documented or altered by prior treatment.

The diagnosis was supported using established classification frameworks, particularly the Yamaguchi criteria, applied within a clinical context. The patient met multiple major and minor criteria, including inflammatory polyarthritis, leukocytosis with neutrophilic predominance, sore throat, and exclusion of infectious, malignant, and alternative autoimmune etiologies. While not all classification elements were contemporaneously documented during hospitalization, the overall pattern remained strongly suggestive of AOSD.

In this case, the patient demonstrated multiple features consistent with a systemic autoinflammatory process, including persistent fevers by history, inflammatory polyarthritis with objective synovitis, leukocytosis with neutrophilic predominance, transaminitis, and markedly elevated inflammatory markers. Antinuclear antibody and rheumatoid factor testing were negative, and an extensive evaluation failed to identify an infectious, malignant, or alternative autoimmune explanation for his presentation. Although documented inpatient temperatures did not reach the ≥39°C threshold often cited in classification frameworks, daily fevers were reported prior to admission, and the overall clinical pattern remained strongly suggestive of AOSD.

Marked hyperferritinemia, while not part of formal classification criteria, further supported the diagnosis in this case. Extreme elevations in ferritin have been well described in AOSD and reflect severe systemic inflammation. When interpreted alongside compatible clinical features and exclusion of competing diagnoses, hyperferritinemia serves as an important supportive diagnostic feature.

CPPD crystals as a competing explanation

The detection of CPPD crystals on knee aspiration introduced diagnostic uncertainty. CPPD can cause acute inflammatory arthritis and may be associated with elevated inflammatory markers during flares [14]. In this case, the presence of synovial fluid crystals provided a plausible explanation for focal joint inflammation. However, it did not account for the patient’s diffuse polyarthritis, persistent fevers, or extreme elevation of inflammatory markers. The overall pattern suggested a process beyond isolated crystal arthropathy. Localized findings, even when objectively confirmed, must be interpreted within the broader clinical context to avoid premature diagnostic closure. This principle is especially important in younger adults, in whom CPPD is less commonly encountered and may represent a coincident or secondary finding rather than the primary driver of disease.

Hyperferritinemia and evaluation for MAS or HLH

Ferritin elevation is a well-recognized feature of AOSD and can reach extreme levels [10]. Marked hyperferritinemia appropriately raises concern for MAS or HLH, both of which may complicate autoinflammatory disease and require prompt recognition [11]. In this case, evaluation proceeded with bone marrow biopsy due to ferritin levels exceeding 20,000 ng/mL.

However, the absence of cytopenias, normal triglyceride levels, and lack of hemophagocytosis on biopsy lowered the likelihood of MAS or HLH. This highlights the challenge of balancing vigilance for life-threatening complications with proportional diagnostic testing. While no single laboratory value should dictate invasive evaluation, extreme ferritin elevations should prompt careful consideration of MAS, particularly when accompanied by cytopenias or rapid clinical deterioration [11].

Therapeutic considerations

Treatment of AOSD typically begins with systemic corticosteroids, which remain effective for rapid control of inflammation [3,12]. Nevertheless, many patients require additional therapy because of incomplete response or steroid dependence. Biologic agents targeting key cytokines, particularly IL-1 and IL-6, have demonstrated high efficacy in controlling systemic features and improving long-term outcomes [5,6,13].

In this case, the patient’s rapid defervescence and improvement in joint symptoms following initiation of high-dose corticosteroids supported an inflammatory rather than infectious etiology. Ongoing disease activity prompted escalation to IL-1 inhibition, with transition from anakinra to canakinumab in combination with methotrexate, resulting in marked clinical improvement. While the therapeutic sequence followed established management strategies, it illustrates how treatment decisions were guided by clinical response in the setting of diagnostic uncertainty.

Broader implications

AOSD continues to impose a substantial disease burden, including impaired quality of life and increased healthcare utilization, particularly in patients with delayed diagnosis or refractory disease [9]. This case highlights several important teaching points. AOSD should be considered in patients with persistent fever and systemic inflammation once infection, malignancy, and other autoimmune conditions have been reasonably excluded. Marked hyperferritinemia, while not included in formal classification criteria, remains a valuable supportive feature when interpreted in a clinical context. Finally, systemic corticosteroids and IL-1-targeted therapies can be highly effective for both induction and long-term disease control in patients with systemic AOSD. Maintaining a broad differential diagnosis remains essential when evaluating patients with fever of unknown origin and systemic inflammation, even when partial explanations emerge during the diagnostic process.

## Conclusions

AOSD is a clinical diagnosis that should be considered in patients with persistent fever and systemic inflammation after infection, malignancy, and other autoimmune conditions have been reasonably excluded. This case highlights how partially explanatory findings, such as calcium pyrophosphate crystal detection, may complicate diagnostic reasoning when the full systemic presentation is not accounted for. Marked hyperferritinemia, while not included in formal classification criteria, can provide an important supportive clue when interpreted in a clinical context. Early recognition and appropriate immunomodulatory therapy, including corticosteroids and IL-1-targeted agents, may lead to significant clinical improvement. While the single-patient nature of this report necessarily limits conclusions, the case reinforces the importance of maintaining a unifying diagnostic framework when evaluating patients with systemic inflammation.
